# Transient obturator nerve block with fascia iliaca compartment block following local infiltration for open inguinal herniorrhaphy

**DOI:** 10.1186/s40981-018-0200-3

**Published:** 2018-08-31

**Authors:** Takeshi Murouchi

**Affiliations:** Department of Anesthesiology, Asahi General Hospital, I-1326, Asahi, Chiba, 289-2511 Japan

**Keywords:** Transient femoral nerve palsy, Ilioinguinal nerve block, Inguinal herniorrhaphy

## Abstract

**Background:**

Ilioinguinal nerve block is effective for analgesia after open inguinal herniorrhaphy. However, transient femoral nerve palsy can happen after the block. The ambulatory patients sometimes cannot discharge from the hospital with the motor deficiency. Here is described a case of fascia iliaca compartment block with complete obturator nerve block after surgical infiltration analgesia for open inguinal herniorrhaphy.

**Case presentation:**

An ambulatory open inguinal herniorrhaphy was performed for a 63-year-old male under general anesthesia. The mixture of short-/long-acting local anesthetics was injected by the surgeon into the subcutaneous tissue, between the Camper’s and Scarpa’s fasciae, into the inguinal canal, and between the internal oblique and transversus abdominis muscles. The patient could not adduct his ipsilateral hip joint at all 1 h after emergence. The flexion of the hip joint was weakened two more hours later, and numbness of the lateral thigh emerged. The complications completely resolved 7 h after surgery.

**Conclusions:**

Surgical infiltration analgesia as well as percutaneous ilioinguinal nerve block can cause both fascia iliaca plane block and obturator nerve block. Analgesia regimen should be carefully built for ambulatory surgery.

## Background

Transient femoral nerve palsy is a known complication after ilioinguinal/iliohypogastric nerve block. Here is described a weak fascia iliaca compartment block (FICB) with complete obturator nerve block (ONB) after local infiltration for open inguinal herniorrhaphy.

## Case presentation

Written informed consent was obtained from the patient for the publication of this case report.

An otherwise healthy 63-year-old man (166.7 cm/54 kg) with left inguinal hernia was planned to undergo an ambulatory open herniorrhaphy. General anesthesia combined with local infiltration was planned, according to the rule of the hospital for ambulatory herniorrhaphy.

Premedication was not given. Standard monitors were attached to the patient on entering the operating room. Anesthesia was induced with 140 mg of propofol, and 50 mcg of fentanyl after the venous access was established. The airway was secured with supraglottic airway after muscle relaxation had been acquired with 0.9 mg/kg of rocuronium. Anesthesia was maintained with 5% desflurane, and mechanical ventilation by pressure control was maintained throughout the surgery. The local anesthetic mixture was made from 10 mL of 0.75% ropivacaine and 10 mL of 1% lidocaine with adrenaline 1:200,000. The surgeon administered 5 mL of the mixture into the subcutaneous tissue before the incision, 3 mL between Camper’s fascia and Scarpa’s fascia, and 2 mL into the inguinal canal. The rest of the mixture (10 mL) was administered with intense pressure between the internal oblique and transversus abdominis muscles at the end of the surgery before skin closure. Another 50 mcg of fentanyl was administered intraoperatively, and 0.5 mg of droperidol was administered before emergence. Surgery was performed with only slight changes in heart rate and blood pressure. The muscle relaxation was reversed with 200 mg of Sugammadex at the end of the surgery. The patient was extubated after stable spontaneous respiration, and good emergence was confirmed. The patient seemed to be able to perform the adduction and abduction of his shoulders, and the extension and flexion of his elbows, hips, knees, and ankles at this point. The duration of the surgery was 45 mins, and anesthetic time was 70 mins. The patient was transferred to the postanesthetic care unit without trouble.

The patient condition was observed 1 h after the transfer. The mental status was alert with E4V5M6 at Glasgow coma scale. He experienced no postoperative nausea and vomiting. There was a slight dull pain around the surgical wound, which soon resolved after 1000 mg of rescue acetaminophen. He was able to drink clear water without nausea or aspiration. Although the patient was able to walk to the restroom with the drip stand and void without trouble, he complained of discomfort in his left thigh. The physical examination revealed that we had not recognized that he could adduct his left hip joint poorly with grade 1 at the manual muscle test (MMT) without any apparent sensory deficit in the left leg. His leg flexion, knee extension, and the sensation of the lateral and medial thigh were normally maintained. Three hours after the surgery, the adduction of the hip recovered to MMT 3. However, the patient could not perform ipsilateral leg flexion nor knee extension against moderate pressure (MMT 4), and there was slight hypoesthesia in the lateral thigh. Seven hours after the surgery, both the muscle weakness and hypoesthesia completely resolved. The patient had not experienced any pain after the first rescue. The patient strongly wished to discharge from the hospital and discharged without trouble.

## Discussion

Transient femoral nerve palsy (TFP) is a rare complication after ilioinguinal nerve block [1]. The complication had been reported after both of percutaneous block [1–3] and surgical field block [4]. The incidence of TFP is reported to be around 3–5% in pediatric population [2]. However, there had been no previous report of fascia iliaca compartment block with obturator nerve block as observed in this case.

The mechanism of this complication is considered as multifactorial. The cause of complete ONB, in this case, was considered due to the intraabdominal dissemination of the local anesthetic mixture after the peritoneal dissection around the pubis. The obturator nerve, along with the obturator artery and vein, runs toward the superior pubic ramus and pierces the obturator internus muscle. The injected local anesthetic was estimated to infiltrate into the abdominal cavity and affect the obturator nerve, adjacent to the incision at this point. The mechanism of FICB is considered to the spread of the local anesthetic toward the lateral edge of the psoas major muscle between the transversus abdominis muscle and the continuum of the transversalis fascia/fascia iliaca [5]. Ultrasound has also revealed that a large volume of the local anesthetic can spread into the femoral triangle [4]. The slow and incomplete effect of the FICB in this case could be the result of the relatively small amount of the injected local anesthetic. Three hours after the administration of the local anesthetic is extraordinarily late for the onset of a block. The frequent active movement of the hip joint by the patient after he noticed the muscle weakness of the adductors could have facilitated the spread of the small amount of local anesthetic toward the psoas major muscle.

The utilization of ultrasound guidance for percutaneous ilioinguinal nerve block was reported to decrease the incidence of TFP [6]. It is also estimated that smaller amount of the local anesthetic should be administered for the prevention of inadvertent spread toward the femoral triangle or the psoas major muscle. The injection of local anesthetic after the surgical incision of the related fasciae should be avoided to prevent the inadvertent intraperitoneal spread as in this case. After this case, our hospital has changed the analgesic regimen from the intramuscular injection to subcutaneous dissemination before the wound closure. To our knowledge, an ONB with FICB after local infiltration for open herniorrhaphy has not been reported. Although both regional anesthesia and local infiltration technique are mandatory for reduced opioid usage, it is also essential to build an effective, but motor-sparing analgesic regimen for ambulatory surgery.

## Abbreviations

FICB: Fascia iliaca compartment block
MMT: Manual muscle test
ONB: Obturator nerve block
TFP: Transient femoral nerve palsy
